# Giant cell tumour of the larynx—a diagnostic challenge

**DOI:** 10.1093/bjrcr/uaaf006

**Published:** 2025-02-10

**Authors:** Vitor H Fraga de Abreu, Ricardo Pacheco, Fernando Cunha, Alexandra Borges

**Affiliations:** European Telemedicine Clinic SL, Neuroradiology Section, 08005 Barcelona, Spain; 2, Otolaryngology Department, Portuguese Institute of Oncology Francisco Gentil, 1099-023 Lisbon, Portugal; Pathology Department, Portuguese Institute of Oncology Francisco Gentil, 1099-023 Lisbon, Portugal; Radiology Department, Portuguese Institute of Oncology Francisco Gentil, 1099-023 Lisbon, Portugal

**Keywords:** giant cell tumor, larynx, imaging, CT

## Abstract

Giant cell tumours (GCTs) of the larynx are extremely rare. The most affected structure is the thyroid cartilage and only 44 cases are reported in the literature.^1^ Clinically, their first manifestation is usually a palpable neck mass, often accompanied by hoarseness, dyspnoea, and/or dysphagia depending on size and location. GCTs are benign neoplasms, yet awareness of this entity is crucial as their aggressive local behaviour can cause significant morbidity with airway obstruction and can simulate a malignancy. The differential diagnosis is broad and remains a clinical and radiological challenge. Indeed, the final diagnosis is established by histology postoperatively. Surgery is the preferred treatment but may impair voice quality. For incomplete surgical resections and high surgical risk lesions, denosumab treatment is a valuable option. We share our experience on GCTs in a young adult presenting as a supraglottic mass.

## Clinical presentation

A man in his 30s was referred for further investigation of a palpable right-sided anterior cervical mass in the infrahyoid neck. It was identified during the workup of longstanding dysphonia, which had started 6 months ago, after the accidental ingestion of a fishbone. His past medical history was relevant for allergic rhinitis and occasional smoking, though unremarkable for malignancy, prior radiation exposure, and alcohol consumption.

## Investigations

On physical examination, the neck mass was palpable and mobile with swallowing. There were no other masses or palpable lymph nodes. Flexible laryngoscopy revealed a large and pale submucosal mass expanding the right false vocal fold and protruding into the laryngeal vestibule. The vocal cords were mobile and there were no mucosal abnormalities.

Laboratory blood tests were unremarkable, including sedimentation rate, C reactive protein, and thyroid and parathyroid function. Chest X-ray was also normal.

Fine needle aspiration cytology (FNAC) of the cervical mass was performed. The collected smears consisted of moderate cellularity with predominant multinucleated giant cells in a haemorrhagic stroma. There was no cytological atypia. On immunohistochemistry, the giant cells were positive for CD68 and for thyroid transcription factor-1 and pan-cytokeratin monoclonal antibody AE1/3 was negative. These findings were initially deemed compatible with a chronic inflammatory process with giant cells, herein a possible foreign body reaction.

## Imaging findings

**Figure 1.**
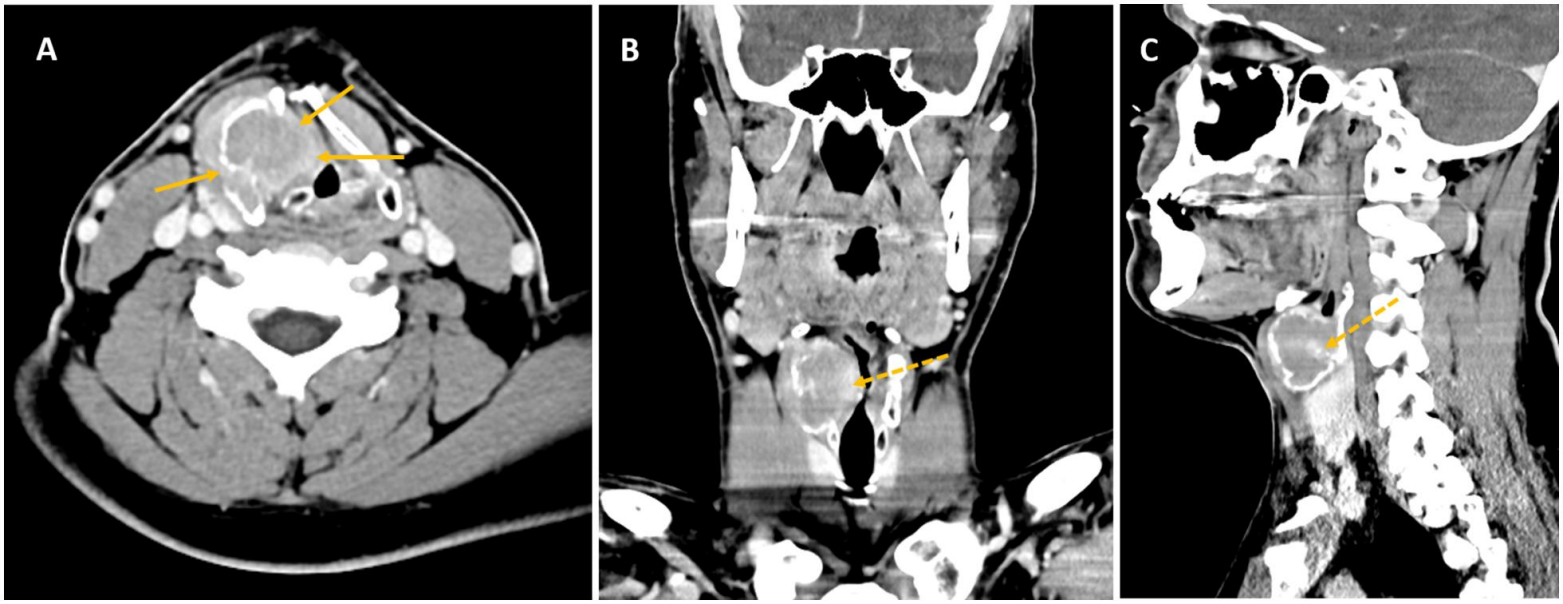
Computed tomography of the neck performed after intravenous iodine contrast administration on axial (A), coronal (B) and sagittal (C) planes show a large predominantly hypodense, lobulated mass expanding, and remodelling the right thyroid lamina with areas of cortical dehiscence more prominent in the inner cortex (solid arrows on A). The mass bulges into the paraglottic space and reduces the caliber of the glottic and supraglottic airway, which is displaced to the left. Cranio-caudally it extends from the inferior margin of the hyoid bone to the vocal process of the arytenoid cartilages. Note the presence of peripheral contrast enhancing soft tissue components (dashed arrows on B and C) and the absence of internal osteoid matrix or chondroid calcifications.

A contrast-enhanced CT of the neck identified a large, well-defined hypodense lesion centred in the right thyroid lamina, leading to smooth cortical expansion, remodelling and dehiscence more striking in the inner cortex of the cartilage, measuring 40 × 33 × 33 mm (extending from the inferior margin of the hyoid bone to the plane of the vocal cords). The lesion (Figure 1) had lobulated contours and showed mild diffuse peripheral contrast enhancement. It caused mass effect upon the adjacent structures, with obliteration of the right paraglottic fat and bulging of the false vocal fold into the laryngeal vestibule decreasing the airway caliber. Bone windows disclosed distinct osteolysis of the right thyroid lamina with no internal calcifications, bone matrix, or periosteal reaction. There were no other lesions of the laryngeal cartilages nor in the laryngeal mucosa, no evidence of radiopaque foreign bodies and no cervical lymphadenopathy.

### Differential diagnosis of giant cell tumour based on imaging findings

A submucosal laryngeal mass in the infrahyoid neck poses a diagnostic challenge. It has a broad differential, including benign and malignant epithelial and non-epithelial tumours, infectious/inflammatory conditions and even direct tumoural invasion from neighbouring structures, such as the hypopharynx, thyroid gland, and lymphadenopathies of the central compartment.

Imaging is helpful in determining the origin and extent of lesions, thus limiting the differential diagnoses. In our case, the lesion could be assigned to the thyroid cartilage, the imaging features and absent locoregional lymphadenopathy favouring a slowly growing, non-aggressive lesion. However, histopathologic confirmation is required as imaging alone cannot exclude malignancy.

When lesions can be assigned to the laryngeal cartilages, such as in our case, benign and malignant chondroid and bone tumours, fibro-osseous lesions and metastases come high in the differential list. Chondroid tumours often show typical “ring-and-arcs” or amorphous “pop-corn-like” matrix calcifications, yet these can be absent in up to 25% of cases.^2^ Although benign and malignant counterparts cannot be accurately distinguished, larger lesions with ill-defined or infiltrating borders should be managed as malignant. Fibro-osseous lesions including ossifying fibroma and fibrous dysplasia affecting the larynx are exceedingly rare. The former manifests as a single, expansile lesion and the latter is usually part of a polyostotic disease. An osteoid matrix with a ground glass appearance affecting the medullary cavity with cortical preservation are key imaging features. As laryngeal cartilage ossifies over time, bone tumours can occur, but these are quite rare with imaging features as elsewhere in the skeleton. Aneurysmal bone cysts (ABC) manifest as expansile osteolytic lesions containing fluid-fluid levels; osteoblastomas as osteolytic lesions surrounded by a shell of cortical bone and containing osteoid matrix; osteosarcomas as expansile, ill-defined, infiltrating lesions with osteoid matrix and a malignant pattern of periosteal reaction (hair-on-end or sunburst) and multiple myeloma as expansile osteolytic lesions with a spontaneously hyperdense soft tissue component showing avid contrast enhancement and restricted diffusivity on diffusion-weighted MR imaging. Giant cell tumours (GCTs) are expansile, lobulated lytic lesions with contrast-enhancing soft tissue components, with no chondroid or osteoid matrix and with low signal intensity on T2W MRI accounting for the presence of hemosiderin and collagen matrix. This feature is useful in the differential diagnosis with chondroid tumours, featured by bright signal on T2W images reflecting the presence of hyaline cartilage. Finally, laryngeal metastases result from hematogenous spread and are rarely the first presentation of a malignancy. The most common primaries are melanoma, renal cell, and bronchogenic tumours. FDG-PET-CT is valuable to depict locoregional and systemic disease in the case of a suspected malignancy. Although the presence of hypermetabolism on fluorodeoxyglucose positron emission tomography (18FDG-PET-CT) is often associated with malignancy, one should be aware that GCTs may be FDG-avid even when benign.^3^

In our case, the clinical, radiological, and FNAC findings suggested a giant cell lesion: either a reparative giant cell granuloma, eventually associated with the prior history of an impacted fish bone, a GCT or a brown tumour of hyperparathyroidism. The latter was excluded because of normal calcium and parathormone levels and absence of parathyroid adenomas on the neck and mediastinal CT scan.

## Treatment

**Figure 2.**
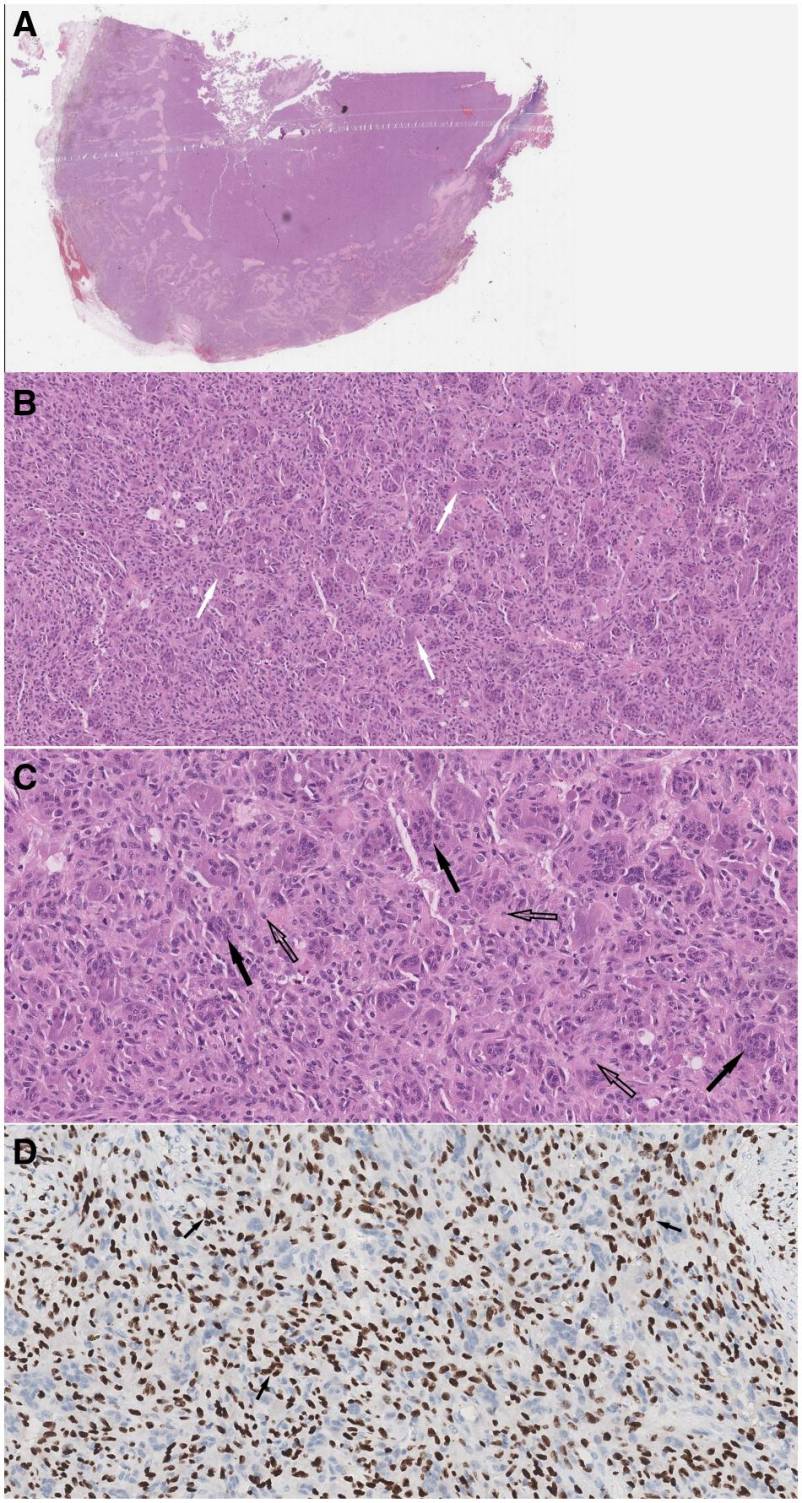
Pathology. Surgical specimen histology. Microscopic slides stained with haematoxylin-eosin (HE) at (A) low, (B) medium, and (C) high power magnifications show a uniformly arranged multinucleated giant cells (dark arrows), reactive osseous metaplasia (white arrows) and a collagenous stroma composed by mononucleated cells with ovoid and fusiform nuclei (open arrows). (D) High power magnification highlights the stromal nuclei which can also be seen inside the giant cells (arrows). Immunohistochemistry shows positive staining for H3.3 G34W in giant cells.

After discussing treatment options with the patient and the multidisciplinary team, it was decided to perform surgery to establish a final diagnosis and alleviate the symptoms. With the abovementioned presumptive diagnoses, the patient underwent a right-sided partial laryngectomy with open tracheostomy. A large expansile lesion was visualized within the whole thickness of the right thyroid lamina. Intraoperative histological analysis disclosed a mesenchymal neoplasm and total resection of the lesion was achieved with sparing of the right arytenoid cartilage. Subsequent histopathological evaluation (Figure 2) of the surgical specimen confirmed a uniformly arranged multinucleated GCT with several foci of reactive osseous metaplasia in a stroma composed by mononucleated cells with ovoid and fusiform bland nuclei without atypical features. There was no evidence of lymphovascular or perineural invasion. The final histopathologic diagnosis was GCT of the bone without malignant differentiation. As the surgical margins were free of tumour, no adjuvant treatment was undertaken.

## Outcome and follow-up

There were no complications intraoperatively. Immediately after surgery, respiratory support through endotracheal intubation and enteric feeding was ensured. The tracheostomy tube was removed 10 days after surgery. On day 12, the nasogastric tube was removed and oral feeding resumed. The patient was discharged 2 weeks after uneventful hospitalization. Regular clinical surveillance without imaging is under course. The patient resumed adequate voice quality and normal swallowing with no episodes of choking. On the last patient’s visit, 16 months after surgery, laryngoscopic examination showed adequate caliber of the laryngeal airway and no signs of tumour recurrence.

## Discussion

In the head and neck region, giant cell tumors (GCTs) are found mainly in the maxillary region^4^ and skull base affecting bones formed by endochondral ossification. The laryngeal skeleton is primarily composed of hyaline cartilage until the second decade of life: thereafter, gradually replaced by bone. Primary GCTs of the larynx are extremely rare. Less than 50 laryngeal CGTs have been reported since Wessely^5^ presented the first case in 1940. CGT is a diagnosis of exclusion after discarding a giant cell reparative granuloma and a brown tumour of hyperparathyroidism.

Presenting symptoms are non-specific and depend on the tumour size, location, and growth rate. In the larynx, the most common are, in decreasing order, a painless anterior neck lump, hoarseness, dyspnoea, and dysphagia. On laryngoscopy, GCTs present as submucosal bulges impinging upon the upper airway. The most common location is the thyroid cartilage, followed by the cricoid and epiglottis. On occasion, GCTs originate in soft tissues,^6^ but this subtype is exceedingly rare.

On histopathology, the diagnosis is made by a combination of giant cells and mononuclear cells of the stroma. These mononuclear cells are predominantly round, oval or polygonal in shape and can resemble normal histiocytes. The nuclei of the stromal cells are indistinguishable from those within the multinucleated giant cells. Typically, the GCT matrix contains numerous, thin-walled capillaries, often with small areas of haemorrhage and areas of cystic degeneration. Cytologic nuclear and mitotic atypia must be absent to rule out a malignant bone-forming tumour. FNAC alone is insufficient for the diagnosis due to misleading sampling of peritumoural inflammatory tissue and because other tumour types may contain giant cells.

On immunohistochemical analysis, giant cells are positive for CD68, a marker for lysosomes, whereas their mononuclear cell component expresses alkaline phosphatase, receptor activator of nuclear factor kappa-B ligand (RANKL) and markers of osteoblast lineage. Conversely, cytokeratins, often identified in epithelial tumours, and thyroid-lineage markers such as PAX8, are absent. The histone H3F3A G34W immunohistochemical nuclear expression is seen exclusively in the mononuclear cell population, whereas CD68 and parathyroid hormone-like peptide (PTH-LP) expression is found in the osteoclast-like giant cells, but not typically in the stromal cells. Mononuclear stromal cells are considered neoplastic, but whether giant cells and histiocytic stromal cells are reactive or neoplastic remains undetermined. Immunohistochemistry for anti-histone H3F3A G34W is highly specific and sensitive for tumours harbouring this mutation.^7^

The recommended treatment for laryngeal GCT is complete excision with clear margins, typically a partial or total laryngectomy. Curettage alone has up to 50% local recurrence rates, which usually occur within the first 3 years after resection.^8^ Indeed, achieving microscopically negative margins is important to prevent recurrence. Neoadjuvant therapy such as radiation therapy and/or chemotherapy is usually unnecessary unless aggressive tumour features are present. The efficacy and safety of external beam radiotherapy are debatable in non-resectable or incompletely resected laryngeal GCT. Secondary sarcomatous transformation is reported in <1% of treated GTCs.^9^

Denosumab is a monoclonal antibody that inhibits the activity of osteoclast-like giant cells via the RANK- RANKL pathway. Histologically, the treatment depletes osteoclastic giant cells with sustained suppression of bone turnover in tumours where lytic bone changes predominate. It has been successfully used as neoadjuvant treatment in recurrent or unresectable GCT.^10^

Post-operatively, it may be difficult to distinguish residual tumour from granulation scar tissue, both on clinical and imaging grounds. A post-operative baseline imaging study should be obtained to facilitate the interpretation of follow-up studies, using the same technique, for at least 5 years. Follow-up is particularly crucial after incomplete surgical resections due to the higher risk of local recurrence and metastases. In our case, at 16 months follow-up, there was no evidence of residual disease or recurrence. The patient tolerates soft foods and is pleased with the quality of his voice. Regular ambulatory surveillance with laryngoscopy is underway.

Submucosal supraglottic laryngeal masses have a broad differential diagnosis and remain a clinical and radiological challenge. As they often have a non-specific appearance, attention must be paid to subtle imaging signs that might help narrow the differential diagnosis. Although extremely rare, GCTs should be considered a differential diagnosis of a laryngeal submucosal mass, since this could have a practical impact on surgical planning. Surgery is the treatment of choice, but it may carry an unacceptable functional prognosis, particularly in large lesions requiring total laryngectomy. Treatment with denosumab is a valuable option as a neoadjuvant treatment for high surgical risk patients and for persistent/recurrent disease. Long-term follow-up should be proposed to patients treated with a partial resection, as the risk of recurrence and/or malignant transformation is not negligible.

## Learning points

- Giant cell tumour (GCT) should be included in the differential of tumours arising from the laryngeal cartilages.
- Clinical and imaging features are insufficient to reach a final diagnosis.
- Key imaging features include an expansile lytic lesion with cystic and contrast-enhancing solid components, with low T2 signal intensity and susceptibility artefacts on MRI.
- Rapid growth, local invasiveness, and lung metastases may occur even in benign GCT.
- Complete surgical resection is the treatment of choice and carries a good prognosis.
- Incomplete resections require long-term follow-up due to high rates of local recurrence and possible malignant transformation.

## Informed consent

Written informed consent was obtained from the patient for publication of this case review, including accompanying images.
